# The image features of emotional faces that predict the initial eye movement to a face

**DOI:** 10.1038/s41598-021-87881-w

**Published:** 2021-04-15

**Authors:** S. M. Stuit, T. M. Kootstra, D. Terburg, C. van den Boomen, M. J. van der Smagt, J. L. Kenemans, S. Van der Stigchel

**Affiliations:** Department of Experimental Psychology, Utrecht University, Utrecht, The Netherlands

**Keywords:** Emotion, Visual system

## Abstract

Emotional facial expressions are important visual communication signals that indicate a sender’s intent and emotional state to an observer. As such, it is not surprising that reactions to different expressions are thought to be automatic and independent of awareness. What is surprising, is that studies show inconsistent results concerning such automatic reactions, particularly when using different face stimuli. We argue that automatic reactions to facial expressions can be better explained, and better understood, in terms of quantitative descriptions of their low-level image features rather than in terms of the emotional content (e.g. angry) of the expressions. Here, we focused on overall spatial frequency (SF) and localized Histograms of Oriented Gradients (HOG) features. We used machine learning classification to reveal the SF and HOG features that are sufficient for classification of the initial eye movement towards one out of two simultaneously presented faces. Interestingly, the identified features serve as better predictors than the emotional content of the expressions. We therefore propose that our modelling approach can further specify which visual features drive these and other behavioural effects related to emotional expressions, which can help solve the inconsistencies found in this line of research.

## Introduction

In any social species, the ability to convey an internal state to nearby members of the social group provides adaptational value. Displaying a particular facial expression is an important way of doing just that. The necessary underlying facial musculature that evolved in non-human primate species is thought to be important for the development of complex social structures^1,2^_._ The adaptive nature of this social ability is thought to result in increasingly pronounced forms of facial expressions. Consequently, facial expressions signalling internal states became more distinctive and, importantly, more prototypical^3–5^. While humans demonstrate the ability to express a multitude of emotional expressions, the general consensus among research into emotional expressions is that humans invariably display six discrete affects: anger, fear, disgust, happiness, surprise and sadness^6,7^. These expressions deviate from the standard facial musculature configuration; the neutral expression. The effects of facial muscular deviation go beyond their effects on the sender of the expression; for instance, faces with emotional expressions attract and hold more visual attention compared to neutral expressions^8,9^.

Not all expressions affect observers equally. For instance, Hansen and Hansen observed search asymmetries between particular combinations of emotional expressions^10^. Participants detected angry expressions faster among happy distractors than vice-versa. Additionally, angry expressions were detected faster than happy expressions among neutral distractors. This behavioural finding, an angry superiority effect which was dubbed ‘the face in the crowd effect’, has been widely replicated since then^11–13^. However, a fair amount of research has also found a seemingly opposite effect: an emotional superiority effect for happy facial expressions^14–16^. Still, others have argued that there are no emotional superiority effects at all and that reports of such effects reflect differences in the mouth area^17^. Specifically, visual search was found to be more efficient for emotional expressions with open- versus closed-mouth expressions. The authors conclude that the state of the mouth alone might be sufficient for explaining differences in search efficiency across happy and angry expression. Consequently, they propose the display of teeth as the primary candidate mechanism for this difference^17^. Note that the influence of teeth is not necessarily about the presence of teeth as objects, but the increase in contrast in the image associated with the presence of teeth^18^.

Both the difference in emotional superiority effects and the effect of displayed teeth may be explained by the low-level image features, such as contrast differences, between the expressions^19,20^. In an attempt to explain the variance found across emotional superiority effects, Savage and colleagues ran a series of experiments using face images from both the NimStim and Ekman and Friesen databases^21–23^. They found a range of both happy- and angry emotional superiority effects as a function of the stimulus set used and concluded that emotional superiority effects are not related to the emotional expressions per se but must be associated with stimulus properties present in the face images. This suggestion was further strengthened by a second set of experiments showing that happy and angry superiority effects depend on the stimulus set used, which remained when faces were presented upside-down^24^. Furthermore, Frischen, Eastwood & Smilek suggest that displaying teeth in emotional expressions produces a detection advantage because of increased contrast in the mouth area relative to a closed mouth^18^. These findings suggest that attention effects towards emotional expressions may be better explained in terms of low-level image features, rather than the emotional content of faces. In fact, previous research has shown that the effective contrast and not the emotional content is relevant for attentional shifts towards emotional faces^25,26^. Moreover, predicting initial eye movements has been successful using models based on low-level image properties^27,28^. This raises questions about what exact properties are relevant and how they relate to emotional faces.

A suitable candidate of such a low-level image feature appears to be the spatial frequency content of an image, defined by luminance variations cycling over different amounts of space. Note that visual sensitivity depends on both the spatial frequencies and the orientations within an image^29^. Face perception may not be equally reliant on all frequencies in each emotion^30,31^. Previous research has shown that identification and recognition for different expressions can depend on different spatial frequencies. For example, while identification of happy facial expressions relies on lower spatial frequency content, identification of sad facial expressions relies on higher spatial frequencies^32^. Moreover, the exact range within lower or higher spatial frequencies that drive emotion identification and recognition varies between emotional expressions^31^. However, as noted by Jeantet and colleagues, the common approach to understanding the relevance of the specific spatial frequency content of faces may have several limitations^31^. In this approach, faces are filtered to contain a specific range of spatial frequencies, which in turn affects the ecological validity of the results. Moreover, since studies vary widely in their ranges of spatial frequencies, their conclusions depend on what is defined as ‘higher’ versus ‘lower’ spatial frequencies^31^.

Another candidate low-level image feature is its local edge orientations: not all oriented edges within a face image are thought to be equally relevant for emotion recognition. For example, horizontal edges are thought to be among the most relevant for recognition^33^. However, this information was based on the Fourier content of the images and as such does not specify to what structure in the face the horizontal edges belong to. A possible solution to this ambiguity is to extract orientation information locally from the images, which is possible with Histograms of Oriented Gradients (HOGs). In recent years, HOGs have been found suitable descriptors of objects in general and faces in particular. While, like spatial frequencies features, HOGs are also based on contrast energy, HOGs represent locally formed orientation descriptors of an image^34^. Note that, relative to spatial frequency content, HOG is highly spatially specific. As such, the HOG features can be used to analyse images with great(er) spatial specificity. Although both HOG and Fourier content reflect contrast energy along different orientations within the images, they are fundamentally different. Specifically, while HOG features are based on Sobel filters and therefore best capture edges, Fourier features are based on waveforms and therefore best capture repeating patterns such as textures. Also, while Fourier content can be transformed back into the original image perfectly using an inverse Fourier transform, this is not possible using HOG features extracted at a single resolution. This shows that the amount of information captured in the HOG features, although highly spatially specific, also comes at a cost of losing much of the information in the original image. In summary, HOG and Fourier content may capture different aspects of the image and we will therefore use both as complementary descriptions.

In the current study, we aimed to better understand emotional superiority effects by examining the low-level image features associated with attracting the initial eye movement between two expressions. We instructed participants to make an eye movement to the first face they perceive when two faces are presented simultaneously. Our assumption here is that the face that attracted more attention would receive the initial eye movement. The unconventional nature of the task is directly related to our analyses, since this simple design allows us to apply a custom feature selection algorithm that aims to find the features of the faces that best predict the participants’ selection. Our main interest is in happy and angry expressions due to previously reported conflicting superiority effects in visual search. However, we also used sad and neutral faces to increase the variance in the features of the images. In contrast to other feature selection and decoding algorithms, decoding will only serve as the tool for finding behaviourally relevant features. In fact, above chance decoding is mainly relevant because it means we can interpret the selected features used for this decoding as relevant to behaviour. An advantage of using basic image information is that both spatial frequency- and HOG-features result in a more detailed representation of a facial expression, one that goes far beyond its emotional content (e.g. angry, happy, sad and neutral). Consequently, these methods enable more sophisticated, data-driven prediction methods for explaining effects based on emotional content.

## Methods

### Participants

A total of 102 participants (17 males), 11 of which were left-handed, were included in this study in return for course credit in the Bachelor Psychology program. The mean age of the participants was 21.09 years (SD = 2.01). The main reason for the large sample size is that for the current study, both in its paradigm and analyses, no comparable material was available. Therefore, we collected data from all participants who applied within a 3-month time frame. All participants indicated normal or corrected to normal vision, no history of visually triggered epilepsy and no colour blindness. All participants signed an informed consent form before commencing the experiment. This form emphasized that data of the participants would be analysed anonymously and that they were free to leave at any time, without giving any form of formal explanation and without losing their course credits (although these would be scaled to the time spent in the experiment). The study was approved by the local ethical committee of the faculty of social and behavioural sciences at Utrecht University. Furthermore, this research was conducted according to the principles expressed in the Declaration of Helsinki.

### Apparatus

The experiment was run on a computer running Windows 7. The monitor used was a linearized 22-inch PHILIPS 202P70/00 with a refresh rate of 85 Hz and dimensions of 2048 × 1536 pixels. To allow for a fast and easy means to perform our task (selecting one out of two faces), eye movements were recorded using an EyeTribe eye-tracker with a 60 Hz sampling rate and an average spatial accuracy of 0.5°. For integration between the EyeTribe and MATLAB, we used a custom solution developed by Edwin Dalmaijer and colleagues^35^. To provide for optimal measurements during eye-tracking, a metal headrest was used to stabilize the participant’s heads. Both this headrest and the seating for this experiment were adjusted in height to allow participants to look straight ahead while sitting comfortably.

### Stimuli

All stimuli were presented via MATLAB 2016a and Psychtoolbox 3^36–38^. The stimuli used consisted of greyscale photographs of happy, angry, neutral and sad frontal-facing facial expressions with a frontal gaze, with 39 different identities, from the Radboud Faces database (http://www.socsci.ru.nl:8180/RaFD2/RaFD?p=main)^39^. All stimuli were of adult Caucasians. Each trial contained a central circular fixation point (0.6° of visual angle diameter). Two face images were presented 14.6° of visual angle into the periphery in a circular aperture of 14.6° × 14.6° of visual angle (632 by 632 px) at a viewing distance of 57 cm. The size of the faces was matched against the perception of faces in real life situations based on research by Miller^40^.

### Procedure

Prior to the experiment, participants were given both written and spoken instructions concerning the procedure of, and instructions for, the experiment. Next, the EyeTribe was calibrated for the participant. This procedure was repeated after each break in which the participant left the experimental environment. Breaks were built in once every 125 trials (4–7 min). It was up to the participant’s discretion to either take a break or continue with the experiment. At the start of each trial, a grey background was presented with a pseudo-random duration between 500 and 1500 ms. Next, a fixation point was added to the centre of the screen. This fixation point was presented until the participant’s gaze was registered at this location by the EyeTribe. Subsequently, two images of faces with emotional expressions (angry, happy, sad or neutral) were presented on both the left and right side of the screen. Participants were instructed to make an eye movement to the first face they perceived. As soon as the gaze-location overlapped with one of the two presented faces, the trial was ended and the faces were removed from the screen. Note that the motivation for using eye movements as a means for selection instead of manual responses is to allow participants to make a rapid and natural response. The faces were always from different identities and could also be different in their displayed expression, resulting in a total of 16 conditions (4 possible expressions on the left side of the screen times four possible expressions on the right side of the screen). Conditions were counterbalanced for stimulus location and emotional content and each condition was presented 56 times. To aid compliance with the task, the experiment also contained additional trials which were identical to the trials described above with one exception, there was a temporal offset (between 34 and 134 ms) between the presentations of the two faces. Each of the conditions was presented 19 times, resulting in a total of 1120 trials for the full experiment.

### Feature extraction and labelling

**Figure 1:**
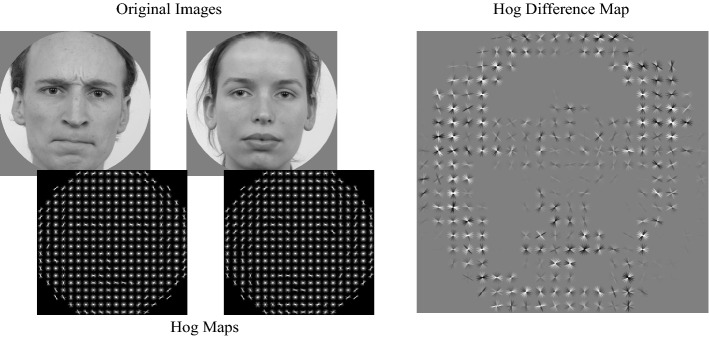
Visualisation of the Fourier feature extraction showing an example of two images used in a trial, their respective, down sampled Fourier features and the Fourier feature differences map. Note that in the Fourier Maps each location corresponds to a particular combination of a spatial frequency and an orientation. The Fourier maps are rotated 90° such that all have horizontal edge contrasts along the horizontal axes, and vertical edge contrast along the vertical axes. The radial axes are for cycles per image (abbreviated to cpi in the figure; ranging from low in the centre to high near the edges). Luminance intensity, from black to white, indicates the relative strength of the contrast for the corresponding section of the map. The Fourier feature differences map was calculated by subtracting the down sampled Fourier features of the image presented on the right, from those of the image on the left. Note that the feature difference map is scaled such that dark regions indicate negative values and light regions indicate positive values.

Spatial frequency information was estimated using the Fourier Magnitude Spectrum (Spatial Frequency, SF). Note that the magnitude spectrum is not informative of the spatial position of a particular contrast. The Fourier Magnitude Spectrum was subdivided into 24 spatial frequencies and 16 orientations, with the magnitudes summed for each oriented SF, resulting in 384 features describing the contrast energy in the images for different SFs and orientations (Fig. 1). Note that magnitudes for frequencies higher than the minimal Nyquist frequency (the Nyquist frequency for the cardinal axes) are excluded. Note that the visualisations of the Fourier magnitude spectra throughout the manuscript are rotated 90 degrees such that magnitudes along the vertical axis reflect contrast energy in vertically oriented edges.

**Figure 2:**
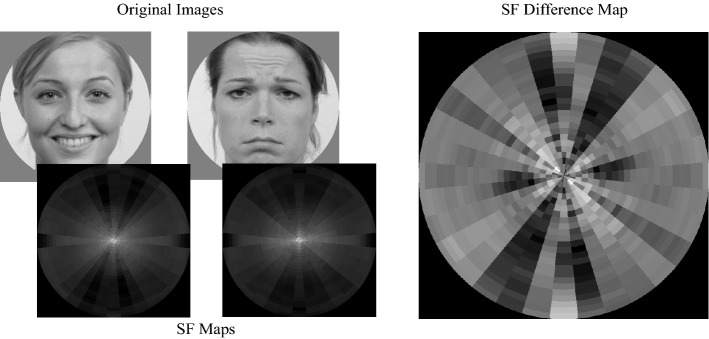
Visualisation of the HOG feature extraction showing an example of two images used in a trial, their respective HOG features and the HOG feature differences map using the highest resolution (10 × 10 cell size). All HOG maps use the same x and y axes as the original images, meaning position in the HOG map is directly coupled with position in an image. The HOG maps show 20 × 20 grids where each position in the grids represents an area of 10 × 10 pixels. For each 10 × 10 pixel area in an image, the weights for 9 differently oriented gradients are calculated. The 9 weights are visualized by white bars where the length reflects the weights. The 9 bars are then superimposed on the 10 × 10 pixel area there are based on. The HOG feature differences map was calculated by subtracting these HOG features weights of the image presented on the right, from those of the image on the left. Note that the feature difference map is scaled such that dark regions indicate negative values and light regions indicate positive values.

For HOG, we subdivided the images into 10 × 10 px non-overlapping sections (Fig. 2). For each section we extracted the power in 9 orientations, resulting in a total of 3600 features describing the spatial orientation structure of the image. Of these, 1116 were excluded from the analysis since they represented sections of the images outside of the aperture used when presenting the face images, resulting in a total of 2484 HOG features used in our analyses. Since the spatial resolution of the HOG features (their cell size) affects what structural components of images are represented, which in turn may affect performance, we extracted two additional sets of HOG features. One with a 20 × 20 px cell size which, after excluding those that corresponded to locations outside of the aperture, resulted in 540 HOG features. The third set used a 40 × 40 px cell size which, after excluding those that corresponded to locations outside of the aperture, resulted in 81 HOG features.

Since we used two faces next to each other in each trial, we subtracted the feature values of the left images from the feature values of the right image (Figs. 1, 2). If the right image received the initial eye movement, this trial was labelled as a 1, if not it was labelled as a 0.

### Data splitting and cross-validation

After feature extracting and labelling the data for a participant, the full data set was divided into 8 partitions, with each partition containing approximately the same balance between the two classes (class 1: left image received eye movement, class 0: right image received eye movement) of trials (those labelled as 0 and those labelled as 1), for eightfold cross-validation. To avoid any possible temporal order effect, each partition contained trials from throughout the experiment. For each fold, one partition was set aside for cross-validation (referred to as the hold out set), the other 7 were used for feature selection. During the feature selection procedure, these seven partitions were further split up into train sets and feature selection validations sets.

### Initial feature inclusion

The feature selection algorithm used aims to create a pool of features for each participant where each feature in the pool can be assigned a weight to reflect its importance. Those weights can then be averaged to describe their overall relevance for our task. The algorithm is a custom-built algorithm combining a filter method and a wrapper method^41^. Note that, at each step of the feature selection (FS) procedure, that is every time features were tested for their additive value for prediction, we pseudo randomly selected approximately 50% of the data as a train set with equal representations of each class (selection of the left versus the right face). The residual data, roughly 50% of the data from the 7 partitions, was used as a validation set to obtain indicators of the relevance of each tested feature. Data from the hold out set was not part of this subdivision and was only used for cross-validation after FS was complete.

**Figure 3:**
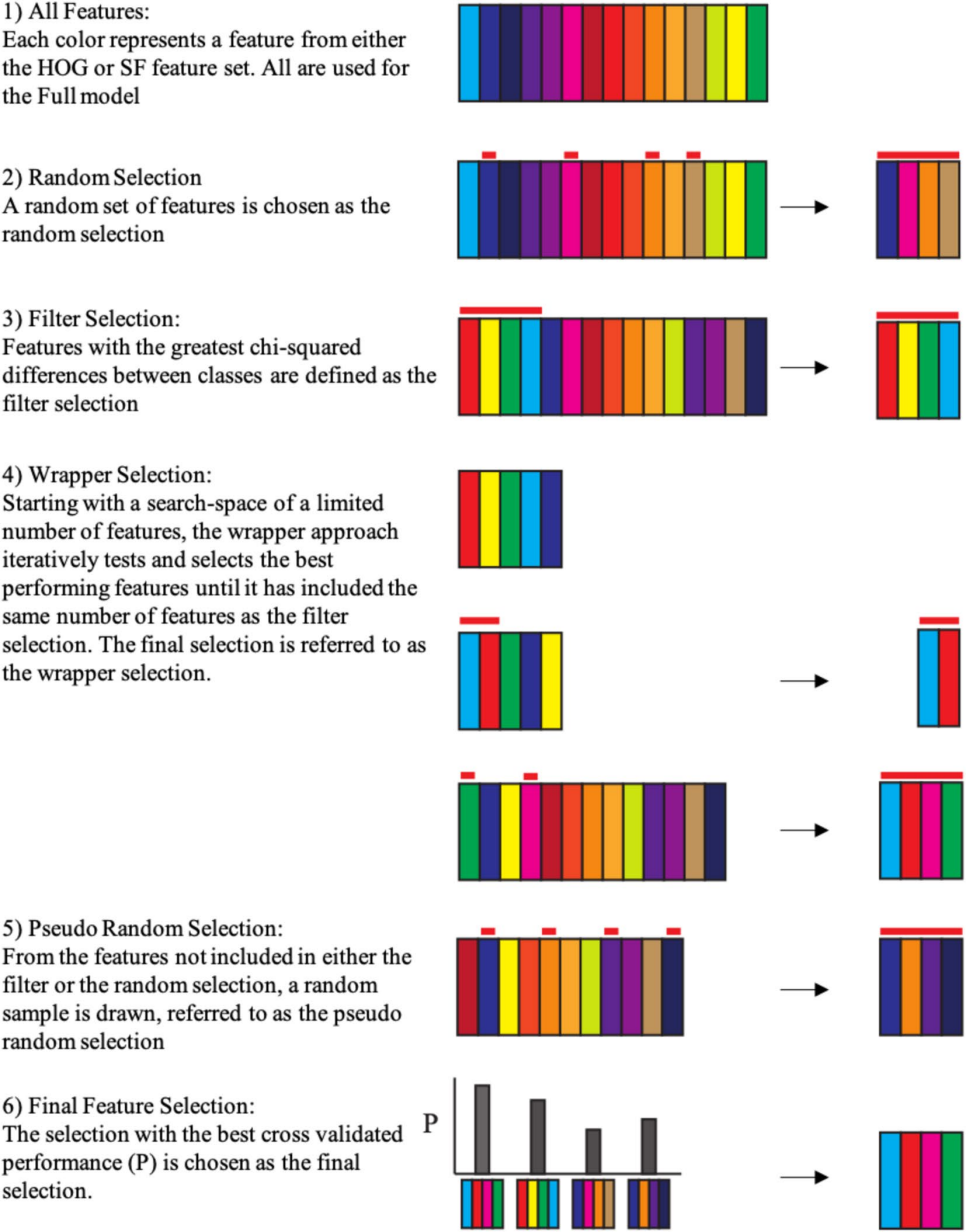
Schematic representation of the feature selection algorithm used in the current project. (1) Visual representation of the feature set. As a comparison to feature selection performance, all of the available features will be used to train and test a model referred to as the Full model. (2) A random collection of features (indicated by the red bars) is selected for a random selection. (3) The features are ranked based on Chi-Square scores. The top of the ranking is used to determine the filter model. (4) A search space is defined from the top-ranking features and the features in this search space are tested for inclusion into the wrapper selection through an iterative process until enough features have been selected for the wrapper model. (5) From the residual features, unused by the wrapper or filter selections, a random selection is made for a pseudo random selection. (6) Each of the four combinations of features are used to train classification models and cross-validation performance is subsequently estimated using the hold-out data. The final selection is based on highest cross-validation performance (P). See the above section Feature Inclusion, and the below section final feature selection for additional details.

For each fold, we first rank each feature based on its chi-squared statistic using Kruskal–Wallis analysis of variance (Fig. 3 step 3). Next, the algorithm uses an iterative wrapper approach to create a selection of features based on each feature’s classification performance, using a linear support vector machine (SVM), in a stepwise additive manner (Fig. 3 step 4). The features that are tested are a subsample of all available features, based on the current ranking. Using the validation set, performance associated with the tested features is ranked based on their F1 score (a measure of accuracy taking into account true positives, false positives and false negatives) since, unlike any training set, the validation set does not necessarily contains an equal representation of trials with each of our labels. The best performing features are selected for inclusion. Note that the ranking for selection for subsequent testing is continuously updated based on the validation performance. This process repeats until the maximum number of features is included, set individually for each fold of each participant. There is, however, one additional step: when 25% of the maximum number of features has been included, the currently included features are all tested separately to see if their inclusion is associated with increased performance. Features that do not aid the performance are excluded. The maximum number of features is based on the initial chi-squared ranking; this is the minimum number of features required to collectively contain 10% of the total sum of chi-squared scores of all features in the analysis. In other words, very high chi-squared values found in the initial ranking result in a lower number of selected features. Our assumption here is that, even though the initial ranking does not take into account interactions between features, higher degrees of separability of the data reflected in the chi-square statistics means that decoding should be possible with fewer features. Note that analyses using HOG and spatial frequency features are done separately.

### Final feature selection

The feature selection algorithm results in four sets of features on which classification models are trained using the current train and validation data and cross-validated on the holdout data. The first selection, referred to as the filter selection (Fig. 3 step 3), uses the features required to collectively contain 10% of the sum of the chi-squared scores of all features. The second selection, referred to as the wrapper selection, uses the same number of features but with the more extensive selection procedure described above (Fig. 3 step 4). The last two selections also contain the same number of features, but here the features are selected either randomly or pseudo randomly. The random selection simply selects a random collection of features for training (Fig. 3 step 2). Note that it is therefore possible, although extremely unlikely, for the random selection to be identical to the filter or wrapper selections. The pseudo random selection, however, takes a random selection of the features that have not been included in either the filter or the wrapper selections (Fig. 3 step 5). These last two selections are included as back up options for when the wrapper or filter selections failed to predict the hold out data well. Our final selection (Fig. 3 step 6), is the best performing, based on the cross-validation accuracy score, of these selections of features since it reflected, empirically, the features that best predict our participants’ behaviour. For comparison, we train one additional model which includes all available features. If all features are relevant for prediction, this model should perform best.

### Estimating chance performance

Based on the procedure for feature selection described above, comparing performance after feature selection with chance is problematic. Specifically, since the performance based on the final selection of features is the highest performance associated with any of the four selections made, the final performance can, theoretically, reach an above chance performance even when all selections perform around chance level, simply because the algorithm always selects based on the highest performance. To overcome this issue, we estimated the empirical chance level under the same conditions as the selection of the final performance. For this estimation, all feature values were shuffled into a random arrangement such that there should be no residual relationship between the class of an example and the associated features. The four models were then trained and cross validated using the shuffled data. From the four resulting cross-validation performances, the highest accuracy was used as empirical chance performance. In this way, the probability to exceed chance level performances in the absence of a relationship between the classes and the features is the same for the final feature selection performance, and for the final control performance, so they can be compared directly. Analysis files are available at https://osf.io/ms8df/.

## Results

### Expression selection behaviour

**Figure 4:**
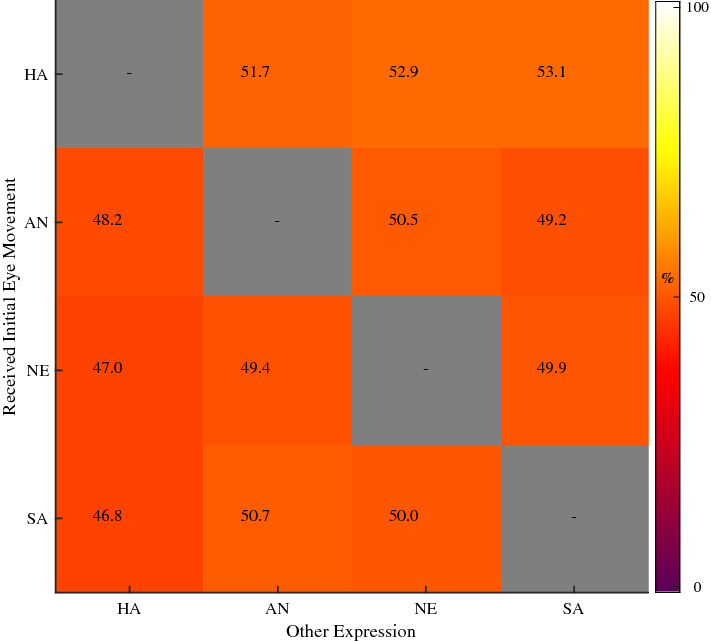
The average percentage of initial eye movements, across participants, towards a particular expression (y-axis; *HA* happy, *AN* angry, *NE* neutral and *SA* sad) separately depending on the expression in the other image (x-axis). Note that this is for trials in which two different expressions were displayed. Results show a small but significant bias towards happy expressions. Figure generated using Matlab 2019b ( www.mathworks.com).

To test for biases in the initial eye movements based on emotional content, we first analysed the initial eye movements for trials where the two faces were presented at the same time and expressed different emotions. We found a bias towards happy faces (Friedman ANOVA, Chi-Squared (3, n = 408) = 14.52, p < 0.01; Fig. 4). These results suggest a happy superiority effect as reported in previous research^14,21,24^. Average median reaction times for trials where dissimilar expressions were presented (Mdn = 0.23 s) did not differ from reaction times where similar expressions were presented (Mdn = 0.24 s; two-sided Wilcoxon Signed-Ranks test, Z = − 1.65, p = 0.10). Finally, consistent with previous reports^42^, we found that 61% of participants were biased to make leftward eye movements. Note that our decoding procedure cannot learn from these biases as it always balances the training to contain equal amounts of data from leftward and rightward eye movements. In the catch trials we see significant differences based on the inter-stimulus-interval (Friedman ANOVA, Chi-Squared (6, n = 606) = 123.19, p < 0.01) where participants are at or above chance in making the initial eye movement to the first presented image for short inter-stimulus-intervals. After 100 ms, participants look at the most recently presented images, suggesting reflexive behaviour. None of the participants were excluded based on the catch-trial data.

### Initial eye movement decoding: different emotional content

**Figure 5:**
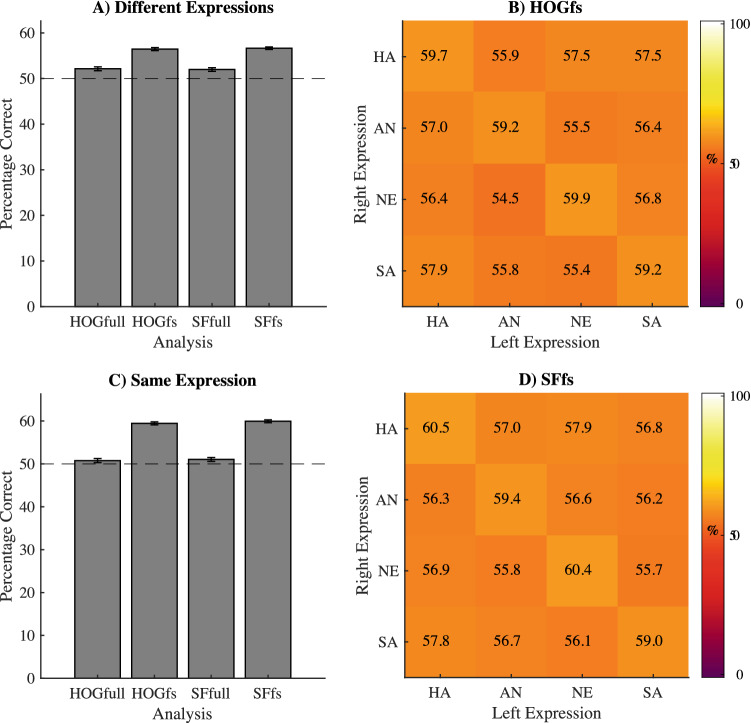
(**A**) The average decoding performance across participants (y-axis) for different modelling procedures (x-axis) based on the trials where different expressions were presented to the participants. (**C**) The same type of results but based on the trials where expressions have the same emotional content. The dotted lines represent the overall empirical chance level performance. Errorbars represent the standard error of the mean. (**B**,**D**) Confusion Matrices for the feature selection models. For all trials of each participant, we reorganized the decoding performance to show how well the model performed for combinations of expressions. Here, performance is represented as a matrix with expression of the right face on the y-axis and left face on the x-axis. Colour intensity reflects the fraction correct for the specific combination of expressions. Note that, performance is nearly equal for all combinations of expressions. Figures generated using Matlab 2019b ( www.mathworks.com).

**Figure 6:**
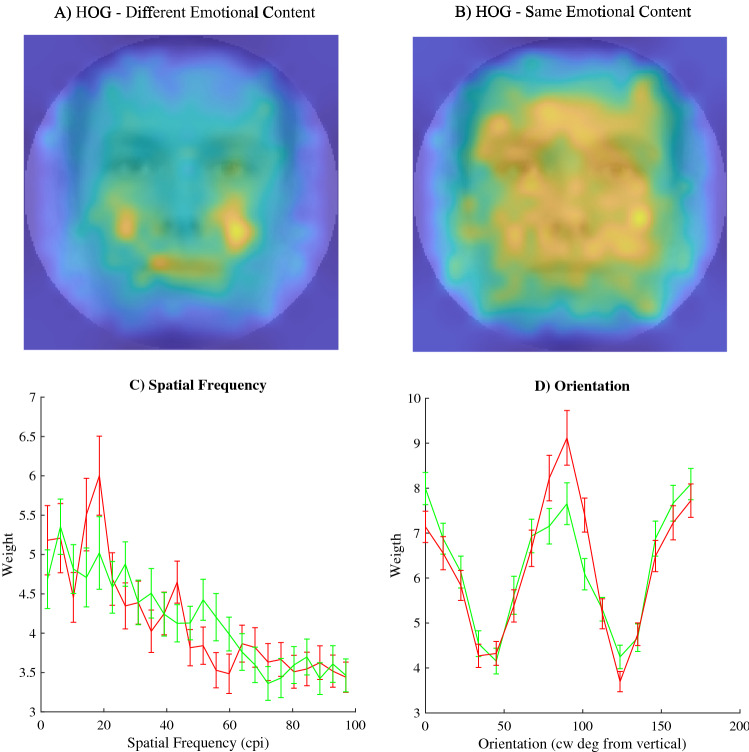
Visual representations of the most relevant features for decoding. (**A**,**B**) Heatmaps (generated using MATLAB 2016a) reflecting the relevance of spatial locations of the HOG features to decoding either trials with different emotional content (**A**) or the same emotional content (**B**) overlaid on the averages of all images with a neutral expression. As relative importance of a location increases, colour changes from blue through green to yellow. (**C**) Here we show the weight, reflecting the percentage of contribution to overall performance, for each band of spatial frequencies used to decode face selection for both trial types (red line, different emotional content; green line same emotional content). Errorbars reflect the standard error of the mean. (**D**) Here we show the weight for each band of oriented edges used to decode initial eye movements for both trial types (red line, different emotional content; green line same emotional content). Errorbars reflect the standard error of the mean. Note that, for both spatial frequency and orientation, the only clear difference is a larger weight for horizontal orientations in trials where the emotional content differs. Figures generated using Matlab 2019b (www.mathworks.com).

To uncover which low-level image features best predict the first face to receive an eye movement, we first looked at the feature selection and decoding results when using only trials in which two expressions with different emotional content were presented. Specifically, we trained the linear SVM using all HOG feature differences (referred to as HOGfull), using a subset of relative HOG features picked by our selection algorithm (referred to as HOGfs), using all SF feature differences (referred to as SFfull) and finally, using a subset of relative SF features as selected by our algorithms (referred to as SFfs). Since HOG spatial resolution (10 × 10, 20 × 20, 40 × 40) did not affect decoding performance for either the HOGfull model (Friedman ANOVA, Chi-Squared (2,n = 305) = 2.44, p = 0.29), nor the HOGfs model (Friedman ANOVA, Chi-Squared (2,n = 305) = 0.31, p = 0.73), performances for the three resolutions were averaged for each participant. Over the 8 folds of the 102 participants, the HOGfs models used 30.23% filter models, 25.29% wrapper models, 23.28% random models and 21.20% pseudo random models. An average of 3.5% of the available features were used in these models. For the SFfs model these percentages were 30.64, 25.12, 22.55 and 21.69%, respectively. The SFfs model used an average of 2.1% of the available features. The relatively high degree of feature inclusion via random and pseudo random selections suggests that the initial rankings on which the filter and wrapper approach are based likely suffer from the high degree of noise within the feature differences in the classes. However, performance was significantly higher when using our feature selection procedure compared to using all low-level image features (Fig. 5A,B; Table 1), indicating the relevance of feature selection. The relevance of different locations was estimated based on the average performances associated with the HOG features and are shown as a heatmap in Fig. 6A. The relevance of different spatial frequencies and orientations, expressed as their contribution to over decoding performances are shown in green in Fig. 6C,D. Results suggest mainly horizontal, low spatial frequencies, and the mouth and cheek areas are used for predicting initial eye movements.

**Table 1:** Statistical test outcome: accuracies different expressions trials.

Comparison	Median(s)	Test	Outcome
HOGfull against control	0.51017/0.50025	Two-sided Wilcoxon Signed-Ranks	Z = 4.145, p < 0.001
SFfull against control	0.50893/0.50	Two-sided Wilcoxon Signed-Ranks	Z = 3.4981, p < 0.001
HOGfs against control	0.5558/0.50025	Two-sided Wilcoxon Signed-Ranks	Z = 8.768, p < 0.001
SFfs against control	0.56399/0.50	Two-sided Wilcoxon Signed-Ranks	Z = 8.7101, p < 0.001
HOGfs against HOGfull	0.5558/0.51017	Two-sided Wilcoxon Signed-Ranks	Z = 8.733, p < 0.001
SFfs against SFfull	0.56399/0.50893	Two-sided Wilcoxon Signed-Ranks	Z = 8.7117, p < 0.001

### Initial eye movement decoding: same emotional content

If initial eye movements are based on low-level image features, a difference in the emotional content should not be required. To test this, we next looked at the initial eye movements when choosing between two faces displaying the same emotional content but with different identities. We again trained four linear SVMs (HOGfull, HOGfs. SFfull, SFfs). Because HOG spatial resolution did not affect decoding performance for either the full model (Friedman ANOVA, Chi-Squared (2,n = 305) = 0.66, p = 0.51), nor the HOGfs model (Friedman ANOVA, Chi-Squared (2,n = 305) = 0.33, p = 0.72), results for the three resolutions were averaged for each participant. Over the 8 folds of the 102 participants, the HOGfs models were based on the filter selection in 30.69% of the folds. The wrapper selection was used in 25.08%, the random selection in 22.59% and the pseudo random selection in 21.36%. An average of 3.5% of the available features were used in these feature selection models. For the SFfs model these percentages were 31.37, 25.12, 23.28 and 20.22% respectively. The SFfs model used an average of 2.1% of available features. As noted above, the relatively high degree of feature inclusion via random and pseudo random selections suggests a high degree of noise within the feature differences in the classes. We found that only the feature selection models, not the full models, resulted in significant decoding performance and again found that performance was significantly higher when using our feature selection procedure compared to using all image-features (Fig. 5; Table 2). The relevance of different locations is shown as a heatmap in Fig. 6B. The relevance of different spatial frequencies and orientations are shown in green in Fig. 6C,D. Results suggest mainly horizontal, low spatial frequencies, and the edges around the nose, cheeks and forehead areas are used for predicting behaviour.

**Table 2:** Statistical test outcome: same expressions trials.

Comparison	Median(s)	Test	Outcome
HOGfull against control	0.50595/0.49702	Two-sided Wilcoxon Signed-Ranks	Z = 1.8142, p = 0.06964
SFfull against control	0.50893/0.50223	Two-sided Wilcoxon Signed-Ranks	Z = 2.2627, p = 0.05
HOGfs against control	0.59524/0.49702	Two-sided Wilcoxon Signed-Ranks	Z = 8.7683, p < 0.001
SFfs against control	0.59821/0.50223	Two-sided Wilcoxon Signed-Ranks	Z = 8.7529, p < 0.001
HOGfs against HOGfull	0.59524/0.50595	Two-sided Wilcoxon Signed-Ranks	Z = 8.7684, p < 0.001
SFfs against SFfull	0.59821/0.50893	Two-sided Wilcoxon Signed-Ranks	Z = 8.6867, p < 0.001

### Decoding the emotional content using the features selected to decode eye movements

**Figure 7:**
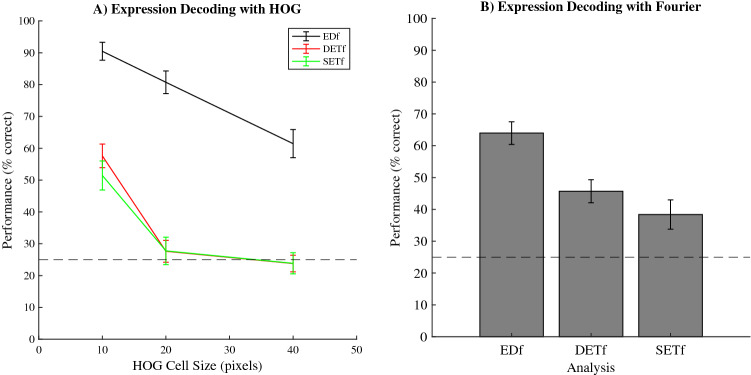
Average decoding performances across all folds (y-axis) based on different sets of HOG and Fourier features. The dotted black lines represent chance level performance. Errorbars represent the standard error of the mean. (**A**) Average performance for decoding emotional content based on HOG features using three different feature sets at three different spatial resolutions (x-axis). EDf (Emotion Decoding features) uses a feature set based on feature selection for emotional content decoding, DETf (Different Emotion Trials features) uses the features based on decoding initial eye movements towards faces with different emotional content and SETf (Same Emotion Trials features) uses the features based on decoding initial eye movements towards faces with the same emotional content. (**B**) Average performance for decoding emotional content based on Fourier features, again using three different feature sets (x-axis). Note that, for both HOG and Fourier features, the features based on decoding initial eye movements are suboptimal for decoding emotional content. Moreover, only the high-resolution HOG features based on decoding eye movement behaviour are relevant for decoding emotional content.

To better understand the image content captured by the selected low-level image features, we tested how well these features decoded the emotional content of the four used expressions (39 images per expression). Specifically, we wanted to know if the low-level image features that predict the initial eye movement are the same features that define, or result in, the emotional content of the expression. If so, our ability to decode may simply reflect the participants’ biases for particular expression. Therefore, we will now focus on decoding emotional content in images, not initial eye movements. Note that the features of the images are now extracted from a single image and do not contain difference scores as for the previous analyses. As such, when we use the same feature, it means the feature will, for example, reflect the same orientation and spatial frequency but only for one image and not the difference between two images. As a reference point for performance, we first used the same feature selection method as used for decoding initial eye movements to estimate performance when the feature selection algorithm had the same degrees of freedom as the feature selection algorithm used to decode initial eye movements in the current experiment. When decoding emotional content, the HOGfs used 4.2% filter selections, 83.3% wrapper selections and 12.5% random selections, using an average of 3.9% of all available features. For expression decoding based on SF features, the feature selection model used 62.5% filter selections and 37.5% wrapper selections with an average of 3.3% of the features. Next, using the exact same folding as used for this emotion decoding, meaning the separations into train and test data were identical, we trained expression decoding models using only the top n features based on decoding initial eye movements in the current experiment. Here, n is based on, and the same as, the number of features used in each fold of the reference’s emotion decoding procedure. While performances for emotion decoding were well above chance level (25%) when the algorithm was free to choose the features used for decoding, emotion decoding using the features found for our behavioural data was below our reference analysis (where the feature selection algorithm decided the features to use for decoding; Fig. 7A,B). Moreover, only the high-resolution HOG features based on decoding initial eye movements are relevant for decoding emotion which is striking since decoding initial eye movements in our task was unaffected by the spatial resolution of the HOG features (Fig. 7A).

### Comparison between predictions based on machine learning with low-level features and emotional content biases

**Figure 8:**
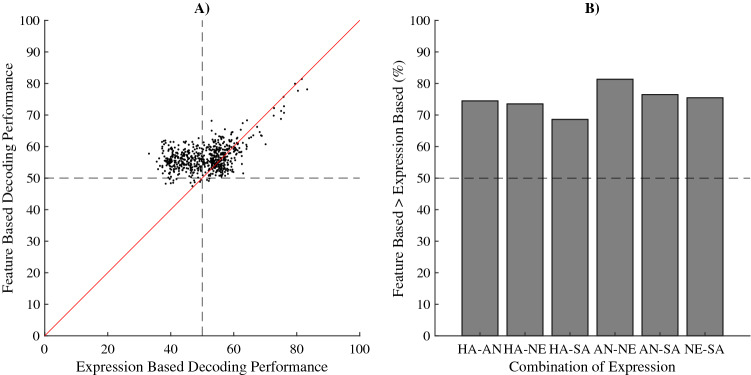
(**A**) Relation between the average percentage correct prediction for each participant and each combination of emotions based on the biases towards expressions (x-axis) and the average corresponding percentage correct prediction based on the low-level image features of the images (y-axis). The dotted vertical line represents chance level performance for emotion-based decoding. The dotted horizontal line represents chance level performance for low-level image features-based decoding. The solid diagonal line shows where performance would be equal. (**B**) For each combination of emotions separately, the percentage of the predictions where low-level image features-based prediction out-performed prediction based on biases towards emotions. Note that for all emotions this is the case (all values well above 50%).

Finally, we evaluated what can best predict the initial eye movement: the emotional content or the differences in low-level image features. Therefore, we aimed to compare the predictive value of our modelling approach to the predictive value based on biases for emotional content seen in the initial eye movements. If our participants simply have a set bias to a particular emotional expression over another in each combination of two emotional expressions, and those biases are the reason we can decode the initial eye movements using low-level visual features, we would expect prediction accuracy based on these biases towards particular emotional expressions to be as large as or larger than the prediction accuracy of our models for decoding initial eye movements. In other words, the maximum decoding performance would be determined by the degree of bias for one emotion over another. However, if the feature differences, independent of the emotional content, provide better insight into the initial eye movements of our participants than the emotional expressions, our models should be better at predicting which emotional expression receives the first eye movement compared to the biases based on the emotional content. To be able to directly compare cross-validation performance of the models (seen in Fig. 5) to the biases seen in the initial eye movements (Fig. 4), we created an additional decoding procedure. For each individual participant, using the same folding as was used for that individual, we determined the bias for one type of emotional content over another (for each emotional expressions pair separately) based on the training data and used that bias to predict initial eye movements in the cross-validation data. The eight resulting performances of each participant for each emotional expression pair were averaged, resulting in one average performance for each pair of emotional expressions. Likewise, we extracted the associated feature selecting decoding performance for each participant and each emotional expression pair. This procedure was performed separately for HOGfs performances and folding and the SFfs performances and folding. The results for the two analyses were averaged and plotted against each other in Fig. 8A. The diagonal line indicates the points where percentage correct for the predictions based on features and those based on biases towards particular emotional content are equal. Note that most decoding performances based on low-level image features are above the diagonal. This suggests that our models were better at predicting the initial eye movement from the differences in the low-level image features than biases for particular emotion seen in the initial eye movements of our participants. In fact, results show that performance based on low-level image features is significantly higher than when based on biases for emotional content (Mdn = 55.95 against Mdn = 52.09 respectively, two-sided Wilcoxon Signed-Ranks test, Z = 15.70, p < 0.001). Furthermore, we also compared performances based on low-level image features and biases for each emotion pair separately (Fig. 8B) and found that only for pairs of happy and neutral faces we can predict initial eye movements based on previous biases (Mdn = 53.09 against chance (50), two-sided Wilcoxon Signed-Ranks test, Z = 2.20, p < 0.001).

## Discussion

We aimed to find the specific low-level image features in emotional expressions that predict the initial eye movement between two emotional expressions. We first show that the initial eye movement between two faces is biased towards happy facial expressions (Fig. 4) compared to angry, sad and neutral faces. More importantly, we found that initial eye movements can be predicted using the differences in either the spatial-structure information (represented with HOG features) or the spatial-frequency contrast information (represented by the Fourier Magnitude Spectrum) in the face images (Figs. 5 and 6). Note that initial eye movements could also be predicted for trials where the faces did not differ in their displayed emotions, but still had different identities. The spatial frequency features relevant for decoding behaviour are also sufficient for classifying the emotions in the images used in the current experiment (Fig. 7). For the HOG features however, only HOG features sampled at the highest resolution were relevant for classifying the emotions used in our task, even though HOG spatial resolution did not affect the ability to decode the *initial eye movements* during the behavioural task. This suggests that decoding initial eye movements based on the lower resolution HOG features is not based on features that capture the emotional content. We go on to show that we can predict the initial eye movements better when based on image features than when based on the emotion in the face (Fig. 8). Taken together, these results suggest that low-level image features can serve as better predictors for initial eye movements than the emotional content itself. Crucially, we also show *what* aspects of the images have predictive value in a data driven manner (Fig. 6). As such, our results give insight into what aspects of faces affect the initial eye movements in our current task. However, we deliberately kept our task rather minimalistic to allow for a (relatively straight-forward), feature selection analysis. Although this means that generalisation of our specific findings to other tasks may be limited, we argue this approach should be applied to other visual attention paradigm as well (e.g. visual-search tasks, emotion-interference task, etc.), as it provides the possibility to identify those low-level image features that underlie behavioural effects related to emotional expressions in other tasks. Since we show significant decoding of the initial eye movements, the selected features can be interpreted as relevant to that behaviour. Before we discuss our main interpretations of the results, we will briefly discuss the unconventional nature of our task and approach such that the strengths and limitation can be taken into account.

The task designed for the current experiment was made specifically for the current feature selection procedure. Since it resulted in a single eye movement based on only two images per trial, the data could be analysed using the current feature selection procedure, something that would not have been possible using a more standard visual search task involving many different faces. The simplistic nature of the task may result in more reflexive behaviour from the participants, in turn favouring predicting via visual features over prediction via emotional content. It is possible that when biases based on emotional content become more obvious, the predictive value based on low-level image features will no longer surpass the predictive value of these emotional content biases. Instead, the predictive value based on low-level image features may start to align with, or even undershoot the predictive value of these emotional content biases. For example, at the extreme of 100% predictable eye-movements based on the emotional content, decoding of the initial eye-movement will be no different from decoding emotional content. Since we see that performance for decoding expressions ranges from between roughly 60–90%, depending on the type of features we use (HOG or SF), a participant’s biases based on emotional content would result in higher performance than predictions based on the low-level image features. However, this would also go against our and others’^25,26^ claims discussed in more detail below that low-level image features are more important than the emotional content. As such, applying the current analytical approach to tasks where biases based on emotional content are very strong will prove highly valuable for this debate. Furthermore, the idea that the initial eye movements in our task is related to attention remains an assumption. Our main aim was to validate a data driven method to find visual features of emotional expressions that predict human behaviour. As such, the current results provide a means to investigate behaviours related to emotional faces in a highly specific, data driven manner.

Still, we argue there are several advantages to the current approach. First and foremost is the level of detail attained. We do not only show biases in the initial eye movements for particular emotions, or what coarse structures (such as the mouth) influence such eye movements, we show which specific low-level image features are relevant for *predicting* these eye movements. For example, the mouth area was previously suggested by Savage and colleagues to be relevant in order to explain inconsistencies between studies reporting on happy and/or angry superiority effects^21^. However, here we show the effect of the mouth may mostly be indirect. Specifically, we show that the nasolabial folds in the cheeks appeared to be more important than the mouth itself. Note that, when expressions with emotional content are presented to our participants, the results show that the relevant features are much less focussed on any specific area of the faces. This suggests that when the differences between the two faces are smaller, participants base their initial eye movements on differences throughout the face. Other approaches, such as filtering images^32,43,44^, eye tracking^18^ and using composite images^11,45^ have not yet attained this level of detail, nor do they give any indication on predictive value.

Our main consideration comes back to emotional superiority effects. Why does the literature consistently show emotional superiority effects but with inconsistent directions^21,24^? Although we cannot generalize our results in a way that would answer this question directly, we now know that both spatial frequency and HOG differences are relevant for initial eye movements between expressions and that there is a dissociation between prediction via low-level image features and via emotional content. Emotional expressions are inherently visual, and expressions are inherently prototypical^23^. Therefore, even though our results show the low-level image features of an emotional expression are better predictors than its emotional content, a clear dissociation between the emotional content and the low-level image features that form that content is impossible. Here we show that, even though spatial frequency content is down-sampled and phase information is ignored, we can use it to decode the emotional content used in the current experiment. This suggests that spatial frequency and orientation patterns are prototypical for different expressions. Since contrast sensitivity varies with spatial frequency and orientation, and spatial frequency and orientation content are predictive for both initial eye movements between two faces and decoding the emotional content, some faces may simply result in stronger visual signals than others based on their respective spatial frequency content. This explanation is in line with previous research showing that it is the effective contrast of the images, and not the emotional content that is relevant for attracting attention^25,26^. This would also explain why face-inversion does also not reliably remove effects related to expressions^24^. Taken together, these and our findings go against the idea of an early warning system for threatening stimuli^46^ and, like Quinlan who argues a lack of evidence for such a threat advantage^47^, we argue that the stimuli used, and their properties, are of great importance.

Note that the influence of spatial frequency is often accounted for by adding control conditions containing inverted faces with the assumption that the spatial frequency is unaffected by face inversion. However, while this holds for images with perfect vertical symmetry, without perfect symmetry, this assumption is only correct for contrasts in the cardinal orientations. Even though contrast sensitivity for diagonal orientations is relatively low^29^, as is their relevance for predicting initial eye movements in the current experiment, neither sensitivity nor the relevance for prediction should be ignored. Note though, that face-inversion did not influence the ability to decode the emotions used in our experiment (data not shown). That being said, face-inversion does also not reliably remove effects related to expressions^24^. As such, the influence of spatial frequency and face inversion on emotional content*-*based effects needs to be tested empirically to fully disentangle the influence of spatial frequencies from those directly related to emotional content.

At high and medium spatial resolutions, HOG features decode emotional content better than spatial frequency content. These features reflect structural aspects of the images and are therefore more likely to be directly related to human expression recognition. However, with exception of the HOG features selected for decoding selection using the highest spatial resolution, the HOG features found relevant to our task are not able to decode the emotional content of the images used in the current experiment. This is in stark contrast to the effect HOG spatial resolution had on the ability to decode initial eye movements. For behaviour, all spatial scales worked equally well for decoding eye movements. This suggest that we can decode initial eye movements also with HOG features that cannot be used to decode emotions. Moreover, we show we can also decode the initial eye movements between two faces with the same expression. Taken together, this suggests that structural parts of the face, specifically configurations of oriented edges, are sufficient to predict initial eye movements (even more so than the emotional content) and these features are not likely related to specific emotions. Taking this into account, as well as our results concerning spatial frequency features, the inconsistency problem with emotional superiority effects may lie in heterogeneity both within and between the emotional expressions. These inconsistencies may be resolved when taking the low-level image features of each image into account. Our current approach is of course only a first step in that direction.

In conclusion, here we show that subsets of the spatial frequency and HOG feature content of emotional expressions predict initial eye movements between two faces. Our results suggest that such low-level image features of emotional expressions serve as a better predictor compared to the emotional content. We suggest that the current approach allows for a better understanding of how different emotional expressions affect behaviour by focussing on data driven stimulus features rather than course expression category labels that obscure important variations within the faces.
